# Genetically-predicted prefrontal DRD4 gene expression modulates differentiated brain responses to food cues in adolescent girls and boys

**DOI:** 10.1038/s41598-021-02797-9

**Published:** 2021-12-16

**Authors:** Andre K. Portella, Afroditi Papantoni, Antoneta T. Joseph, Liuyi Chen, Richard S. Lee, Patricia P. Silveira, Laurette Dube, Susan Carnell

**Affiliations:** 1 Desautels Faculty of Management, McGill Center for the Convergence of Health and Economics, McGill University, Montreal, QC Canada; 2 Postgraduate Program in Pediatrics, Universidade Federal de Ciencias da Saude de Porto Alegre, Porto Alegre, RS Brazil; 3 Department of Nutrition, Gillings School of Global Public Health, University of North Carolina at Chapel Hill, Chapel Hill, NC USA; 4 McGill Centre for the Convergence of Health and Economics (MCCHE), McGill University, Montreal, Canada; 5 Department of Psychiatry and Behavioral Sciences, Division of Psychiatric Neuroimaging, Johns Hopkins University School of Medicine, Baltimore, MD USA; 6 Department of Psychiatry and Behavioral Sciences, Johns Hopkins University School of Medicine, Baltimore, MD USA; 7 Ludmer Centre for Neuroinformatics and Mental Health, Montreal, QC Canada; 8 Department of Psychiatry, McGill University, Montreal, QC Canada; 9 Department of Psychiatry and Behavioral Sciences, Division of Child and Adolescent Psychiatry, Johns Hopkins University School of Medicine, Baltimore, MD USA

**Keywords:** Cognitive neuroscience, Feeding behaviour, Genetics of the nervous system, Motivation, Neural circuits, Reward

## Abstract

The dopamine receptor 4 (DRD4) in the prefrontal cortex (PFC) acts to modulate behaviours including cognitive control and motivation, and has been implicated in behavioral inhibition and responsivity to food cues. Adolescence is a sensitive period for the development of habitual eating behaviors and obesity risk, with potential mediation by development of the PFC. We previously found that genetic variations influencing DRD4 function or expression were associated with measures of laboratory and real-world eating behavior in girls and boys. Here we investigated brain responses to high energy–density (ED) and low-ED food cues using an fMRI task conducted in the satiated state. We used the gene-based association method PrediXcan to estimate tissue-specific DRD4 gene expression in prefrontal brain areas from individual genotypes. Among girls, those with lower vs. higher predicted prefrontal DRD4 expression showed lesser activation to high-ED and low-ED vs. non-food cues in a distributed network of regions implicated in attention and sensorimotor processing including middle frontal gyrus, and lesser activation to low-ED vs non-food cues in key regions implicated in valuation including orbitofrontal cortex and ventromedial PFC. In contrast, males with lower vs. higher predicted prefrontal DRD4 expression showed minimal differences in food cue response, namely relatively greater activation to high-ED and low-ED vs. non-food cues in the inferior parietal lobule. Our data suggest sex-specific effects of prefrontal DRD4 on brain food responsiveness in adolescence, with modulation of distributed regions relevant to cognitive control and motivation observable in female adolescents.

## Introduction

Adolescence is a key period for the development of eating behaviors that confer obesity risk^1,2^. Early in life, the prefrontal cortex (PFC) begins to form critical direct and indirect connections to distributed cortical and sub-cortical brain regions^3,4^. This process continues apace throughout adolescence, with the PFC among the last brain regions to reach maturity^5–7^. Through its interactions with other brain regions, the PFC regulates multiple cognitive functions with potential relevance for eating behavior and thereby body weight, including behavioral inhibition and flexibility, and motivation and reward processing^8^. Consistent with this, studies have shown that obesity is associated with increased impulsive decision-making and attention bias in response to food cues, as well as with altered patterns of activation across the brain in response to food cues presented within fMRI tasks^9^.

Cortical neuronal maturation and the balance between neural excitation and inhibition during adolescence is hypothesized to be dopamine dependent^10^. The dopamine receptor 4 gene (DRD4) functionally produces inhibitory effects, and is expressed in brain regions implicated in planning, executive function and reward, including frontal cortex^11^. Human studies have found that diminished dopamine inhibitory feedback in DRD4-7R carriers is linked to weaker physiological dopamine signaling compared to non-carriers^12–15^. Other studies have shown associations of genetic variants associated with impoverished dopamine function (TaqI A1 (vs. A2)), with higher food reinforcement and energy intake in adults^16^, greater emotional eating and snack food reinforcement in adult females with obesity^17^, and lower satiety and fullness before a meal in children with obesity^18^.

Our previous work has demonstrated that genetic variation on DRD4 is associated with body weight and eating behavior in children, moderating the influence of the quality of the environment^19–26^. For example, the presence of hypofunctional variant DRD4-7R allele in combination with lower maternal sensitivity during the early postnatal period was associated with higher body weight in early childhood^23^. Extending this work, we have used a novel genomic approach that imputes the gene expression of DRD4 in PFC using individual level genomic information^27^ to find that genetically regulated expression of the DRD4 gene in PFC moderated effects of socioeconomic background on emotional eating in 48-month old children, and desire to drink in 60-month old children^22^. A similar differential susceptibility effect was found in the current adolescent cohort, such that lower predicted PFC DRD4 expression was associated with greater food intake in the satiated state, in the context of lower socioeconomic status^20^. This approach represents a significant addition to existing work by examining specific effects of genetically-driven mechanisms on the biological substrate of interest, in this case brain circuits including and interacting with prefrontal cortex.

The above evidence suggests a role of differential DRD4 expression in PFC in influencing obesity risk by affecting the response of individuals to the environment. Studies in females using fMRI have demonstrated that weaker responses in the brain reward circuitry to imagined and actual intake of palatable foods are more strongly associated with greater future increases in body mass in individuals carrying low functioning variants of dopamine receptor genes, such as the DRD2 TaqIA A1 allele and the DRD47R allele^28,29^. However, the specific effects of genetically regulated prefrontal expression of the DRD4 gene on neural responses to food cues have not yet been examined. We therefore investigated whether predicted PFC DRD4 expression would be associated with altered brain food cue responsiveness in adolescents. Given known sex differences in brain maturation during adolescence^30^ as well as in eating behavior^20^, obesity^23^, and neural responses to food cues^31,32^, likely driven by social environment as well as biological factors^25^, we examined relationships in females and males separately. Since obesity is associated with eating in the presence of satiety^33^, and with reduced neural satiety responses^32^, we examined effects of DRD4 PFC expression on neural food cue responses in the satiated/fed, rather than the fasted, state.

## Methods

### Participants

Adolescents and their mothers were recruited for a study investigating the neurobehavioral basis of obesity and familial obesity risk. Details of this study have been published elsewhere^20,34^ and are also described here. In summary, recruitment was via flyers posted at the Johns Hopkins Hospital in Baltimore, MD and online advertisements. Adolescent-mother dyads were required to speak English fluently. For adolescents, exclusion criteria included: being outside our target age range of 14–18 years old, current diagnosis of a significant health problem (e.g. eating disorder, learning disability), use of medication affecting appetite and body weight (e.g. stimulants, antidepressants), participation in a structured weight loss program, medical contraindications to MRI (e.g. metal implants), and food allergies. For mothers, exclusion criteria included: current pregnancy, and excessive smoking, recreational drug use or alcohol intake.

Potential participants completed a telephone screening. Eligible dyads were then invited for a total of three visits: an initial consultation, a scan visit in a fasted condition and a scan visit in a fed condition (condition of interest). During the initial consultation, informed consent was obtained from the 98 adolescent participants. Adolescents 18 years or over completed informed consent for themselves. For adolescents under 18 years, we obtained informed consent from parents, and adolescents themselves provided assent. Fifteen completed neither test day and were excluded from further analysis. Of the remaining 83 participants, we excluded adolescent-mother pairs whose saliva samples were not collected and had incomplete scan data, resulting in a final analytic sample of 73 adolescent-mother pairs who completed the initial consultation and at least one of the test days (no data were imputed).

This study was approved by the Johns Hopkins University School of Medicine Institutional Review Board and all the procedures followed guidelines and regulation in accordance with the Declaration of Helsinki.

### Initial consultation

#### Anthropometric measures

Body weight and fat percentage were assessed at the initial consultation using a SC-331S Total Body Composition Analyzer (TANITA Corp., Tokyo) to measure body weight and estimates fat percentage via Bio-Impedance Analysis. Height was assessed using a wall-mounted stadiometer after removal of shoes. BMI values (kg/m2) were calculated, and BMI z scores and percentiles derived for adolescents based on Center for Disease Control (CDC) growth charts from 2000^35^. Adolescents under the 85th percentile were classified as normal-weight, adolescents between the 85th and 95th percentiles as overweight, and adolescents at the 95th percentile or above as obese. During the initial consultation, saliva samples were collected from all adolescents using the Oragene OG500 (DNAGenotek, Ottawa, Canada) saliva collection kits. Adolescents completed the Pubertal Development Scale (PDS)^36^. Mothers completed a demographic questionnaire reporting their own and their child’s race/ethnicity.

### Scan visits

#### Overview

For the fed scan visit, participants were instructed to arrive fasted (for at least 4 h). Adolescent participants underwent an MRI scan that included a food cue reactivity (FCR) task. Prior to the scan, participants were given a fixed liquid meal preload of 16 fl oz (480 kcal) of the high-protein drink BOOST (480 kcal, 30 g protein, 12 g fat, 36 g sugar per 16 fl oz), which was consumed 38 ± 11 min before onset of the Food Cue Reactivity task.

### Food cue reactivity (FCR) task

Participants completed an FCR task during the MRI scan. This task was programmed in E-Prime (Psychology Software Tools, Inc., Sharpsburg, Pennsylvania). The paradigm comprised an instruction screen that explained the task to the participants and 2 runs with 45 trials each. Each run included 15 high energy density (ED) food trials, 15 low-ED food trials, and 15 non-food trials, appearing in a pseudorandomized order with no more than 2 stimuli of each category in a row. Each trial lasted 4 s (including stimulus + stimulus rating period) and was followed by a fixation period (central crosshair for 1 s). For each trial, a food or non-food picture was presented in the middle of the screen with a wanting rating question and response options at the bottom of the screen. For food pictures the question read: “*Do you want to eat this right now?*” and for non-food stimuli it read: “*Do you want to use this right now?*” with response options as follows: Not at all, Not really, A little bit, Very much. Participants responded using a button box with two fingers (index and middle finger) on each hand. FCR task wanting ratings for each stimulus were coded from 1 to 4. High-ED food, low-ED food and non-food pictures were colored photos matched on size, shape, contrast, and resolution. High-ED food pictures included items such as a donut, chocolate chip cookies, a cinnamon bun, a slice of pizza, and tater tots (10 sweet and 5 savory items per run). Low-ED food photos included items such as a green apple, mushrooms, cooked peas and carrots, and strawberries. Non-food photos included stationery items and other objects such as post-it notes, a highlighter, masking tape, and gold screws. Verbal ratings for 1) hunger, 2) fullness, 3) desire to eat, 4) thirst, 5) stress, and 6) boredom preceded and followed the MRI scan. These ratings were reported on an analog scale ranging from 0–100, where “0” represented “Not at All” and “100” represented “Extremely”.

### fMRI acquisition

Images were acquired using a 3.0 Tesla scanner (Phillips HealthCare, Best, the Netherlands) with a multi-element 32 channel receiver head coil. Anatomical images were acquired using a T1-weighted 3D magnetization-prepared rapid acquisition gradient-echo (MPRAGE) sequence (TR/TE = 8.0/3.70 ms; flip angle = 8°; 1 × 1x1 mm3 resolution; acquisition matrix, 212 × 172; 1-mm thick slices; field of view (FOV), 212 × 172x150; 150 slices). BOLD-weighted functional images were acquired using single-shot SENSE-EPI (TR/TE = 2500/30 ms; flip angle = 70°; 3 × 3x3 mm3 resolution; acquisition matrix, 84 × 81; 3-mm thick slices; FOV, 256 × 256x141 mm; acquisition of 47 contiguous slices). Slices were acquired in ascending order.

### Genetically predicted prefrontal DRD4 gene expression

DNA was extracted from saliva samples collected at the initial consultation. Expression of DRD4 in prefrontal brain regions was computed using PrediXcan, a prediction method that estimates tissue-specific gene expression based on individual-level genotype data^27^. Genotyping was conducted using the genome-wide Illumina Infinium Multi-Ethnic Global Array (MEGA), with clusters for the SNPs defined using GenomeStudio version 2011.1 and GenTrain 1.0. Quality control on the genotyping calls has been previously described^37^. SNPs were verified for a genotyping rate ≥ 95% and no deviation from Hardy–Weinberg equilibrium (P < 0.001), and minor allele frequency ≥ 0.05, using PLINK^38,39^. After quality control procedures and imputation, which included ascertainment of participant sex, 1,767,525 SNPs were available for use in PrediXcan. To describe the population stratification, we performed principal component analysis using SMARTPCA^40^ on this pruned dataset of genotyped SNPs (with r2 < 0.20, sliding window of 50 and an increment of 5 SNPs).

PrediXcan uses a machine learning approach to generate algorithms that estimate the genetically determined component of gene expression in specific brain regions at the individual level from the subject's genotype. The PrediXcan algorithm was built using a reference dataset from deceased human brain donors, comprising data from the GTEx project (version 7)^41^, GEUVADIS^42^ and DGN^43^, containing both genotype and gene expression levels. The PrediXcan method was executed according to methods available in^27^, using GTEX version 7 frontal cortex eQTL model^41^.

### Socioeconomic status (SES) composite score

To assess socioeconomic environment, we used a combination of variables collected as part of the larger study. Detailed description of the composite score calculation is described elsewhere^20^. Briefly, mothers completed a demographic questionnaire including questions on annual household income, maternal education, food insecurity, perceived resource availability, and receipt of public assistance. To ensure that these five variables reflected the same underlying theoretical structure, and to derive a composite score reflecting multiple dimensions of socioeconomic environment, we conducted a principal component analysis (PCA) with Promax rotation. Each of the variables was standardized and weighted by its factor loading to create a composite score in which a higher score indicates higher socioeconomic status.

### Statistical analysis

#### Behavioral data analysis

Analysis of behavioral data was conducted using the Statistical Package for the Social Sciences (SPSS, version 25.0). Adolescents were divided into two groups representing higher vs. lower predicted prefrontal DRD4 gene expression levels (mean split). Wanting ratings for high-ED foods were averaged to create a single value for each participant. This calculation was repeated for low-ED foods and non-foods. Two-way ANOVAs (sex x DRD4 group) were used to compare between-group differences in continuous variables (age, BMI, BMI z-score, BMI percentile, body fat percent, socioeconomic environment score, FCR task wanting ratings, and internal state verbal ratings). Results were corrected for multiple comparisons with Bonferroni adjustment. Chi-square tests were used to assess between-group differences in categorical variables (pubertal status, race). Results were considered significant at p < 0.05 after Bonferroni adjustment.

#### fMRI data analysis

Analysis of fMRI data was performed using Statistical Parametric Mapping software (SPM version 12; Wellcome Department of Cognitive Neurology, London, UK) in the MATLAB R2017a programming environment. The Artifact Detection Tools (ART) toolbox for MATLAB (Gabrieli Laboratory, MIT, Cambridge, MA) was used to detect global mean and motion outliers in the functional data. A participant’s functional data were excluded if > 20% of all the volumes were tagged as high motion (motion > 3 mm in any direction). Subsequently, for each participant’s run, functional images were slice-time corrected using the median slice as the reference slice, realigned to the mean of the images after the initial realignment, normalized to the MNI-EPI template, and smoothed with a 6 mm FWHM Gaussian kernel. For first-level statistical analysis, we constructed mass-univariate general linear regression models for each participant. The regressors included the task conditions (“high-ED”, “low-ED”, “non-food”, “fixation”) as events of interest and the realignment motion parameters as covariates. Task-related regressors were convolved with the canonical hemodynamic response function (HRF) and a high-pass filter of 128 s was applied. The following contrasts of interest were calculated at the single subject level: 1) high-ED vs. non-food; 2) low-ED vs. non-food. For group-level statistical analysis, we assessed the random effects of task-related activity between groups (high DRD4 vs. low DRD4 groups) using Bayesian posterior inference^44^ applied to the contrast images generated from the first-level analysis, including two principal components reflecting population stratification (PC1, PC2) as covariates in the model. The Bayesian approach is optimal for exploratory whole brain analyses since it infers the posterior probability of detecting the observed group effects given the observed activation map without making strong assumptions about effect size^44^, and therefore does not require adjustment for multiple comparisons and the associated risk of over-correction^45,46^. Per^47^, clusters were reported if they had an effect size Cohen’s d > 0.2, a Bayes factor logBF > 3.0, and a cluster extent threshold k > 10.

## Results

### Baseline characteristics

Participant demographics, anthropometrics and means for behavioral variables for our analytic sample are reported in Table 1. Demographic characteristics (child age, child sex, child race, child BMI z-score, and household income) were similar between the final analytic (n = 73) sample and the entire consented (n = 98) sample. However, maternal education was higher in the analytic sample (64.4% graduated from college) compared with the entire sample (55.7% graduated from college) (p = 0.003). Age, BMI z-score, and SES composite score did not differ significantly by sex or DRD4 group, and no sex by DRD4 group interactions were present. Mother-reported race for adolescents did not differ by sex or DRD4 group. As expected, females had significantly higher body fat percent compared to males (F(1,69) = 25.07, p < 0.001) across both DRD4 groups, and pubertal stage was more advanced in females, with females predominantly at the post pubertal stage, and males predominantly in mid and late pubertal stages (p < 0.001), with no evidence for differences by DRD4 group.

**Table 1:** Descriptive characteristics.

	Low DRD4 group (n = 40)		High DRD4 group (n = 33)		Whole sample (n = 73)
	Mean (SD) or N (%)				
	Female (n = 22)	Male (n = 18)	Female (n = 17)	Male (n = 16)	
Age	16.2 (1.4)	16.0 (1.2)	16.6 (1.0)	16.0 (1.3)	16.2 (1.2)
**Pubertal stage**^**a**^					
Early pubertal stage	0 (0)	1 (5.9)	0 (0)	0 (0)	1 (1.5)
Mid pubertal stage	0 (0)	2 (11.8)	0 (0)	4 (33.3)	6 (9.0)
Late pubertal stage	2 (9.5)	14 (82.4)	0 (0)	7 (58.3)	23 (34.3)
Post pubertal stage	19 (90.5)	0 (0)	17 (100.0)	1 (8.3)	37 (55.2)
**Race/Ethnicity**					
White	8 (36.4)	11 (61.1)	11 (64.7)	12 (75.0)	42 (57.5)
Black or African American	11 (50.0)	6 (33.3)	2 (11.8)	4 (25.0)	23 (31.5)
Asian	2 (9.1)	0 (0)	0 (0)	0 (0)	2 (2.7)
More than one race	1 (4.5)	1 (5.6)	3 (17.6)	0 (0)	5 (6.8)
Other/unknown	0 (0)	0 (0)	1 (5.9)	0 (0)	1 (1.4)
BMI	25.4 (7.4)	24.0 (5.8)	23.3 (4.7)	23.8 (6.0)	24.2 (6.1)
BMI z-score	0.76 (1.04)	0.58 (1.27)	0.40 (1.12)	0.49 (1.38)	0.57 (1.18)
Body Fat %	29.8 (10.2)	18.3 (10.4)	27.4 (8.5)	16.2 (8.8)	23.4 (11.1)
SES composite score	− 0.322 (1.147)	0.139 (0.797)	0.328 (0.869)	0.067 (1.060)	0.028 (0.999)
**Food cue reactivity task wanting ratings**					
High-ED foods	2.51 (0.65)	2.61 (0.52)	2.01 (0.64)	2.75 (0.65)	2.47 (0.66)
Low-ED foods	2.57 (0.42)	2.47 (0.54)	2.27 (0.52)	2.47 (0.53)	2.45 (0.50)
Non-foods	1.61 (0.48)	1.57 (0.57)	1.71 (0.45)	1.64 (0.55)	1.63 (0.50)
**Internal state verbal ratings**^**b**^					
Hunger	34.4 (24.4)	48.9 (31.9)	24.4 (27.2)	44.5 (33.3)^c^	37.9 (29.9)
Fullness	66.5 (26.2)	52.5 (28.3)	65.4 (30.3)	48.1 (32.1)^c^	58.8 (29.5)
Desire to eat	37.0 (29.5)	45.0 (32.0)	30.3 (29.5)	41.6 (35.1)	38.3 (31.2)
Thirst	35.9 (27.8)	42.8 (28.9)	26.5 (25.8)	31.9 (35.2)	34.5 (29.4)
Stress	21.6 (32.4)	19.3 (22.8)	29.4 (29.9)	25.0 (34.0)	23.6 (29.7)
Boredom	54.1 (29.0)	44.2 (31.9)	26.9 (18.9)	34.7 (31.2)	41.1 (29.6)

^a^n = 21f/17 m for low group; 17f/12 m for high group; 67 for whole sample.

^b^Internal State Verbal Ratings collected before the FCR task.

^c^Males reported significantly higher ratings for hunger (p = 0.009) and significantly lower ratings for fullness (p = 0.029) relative to females before the FCR task, independent of DRD4 group.

### Internal state ratings

Males reported significantly higher ratings for hunger (F(1,67) = 7.13, p = 0.009) and significantly lower ratings for fullness (F(1,67) = 4.98, p = 0.029) relative to females prior to the FCR task. No sex differences were found for ratings of desire to eat, thirst, stress and boredom.

Adolescents in the low DRD4 group reported significantly higher ratings for boredom (F(1,67) = 7.64, p = 0.007) relative to the adolescents in the high DRD4 group. No other DRD4 group differences were identified. No sex by DRD4 group interactions were identified for any of the internal state verbal ratings assessed prior to the FCR task.

### FCR task wanting ratings

**Figure 1:**
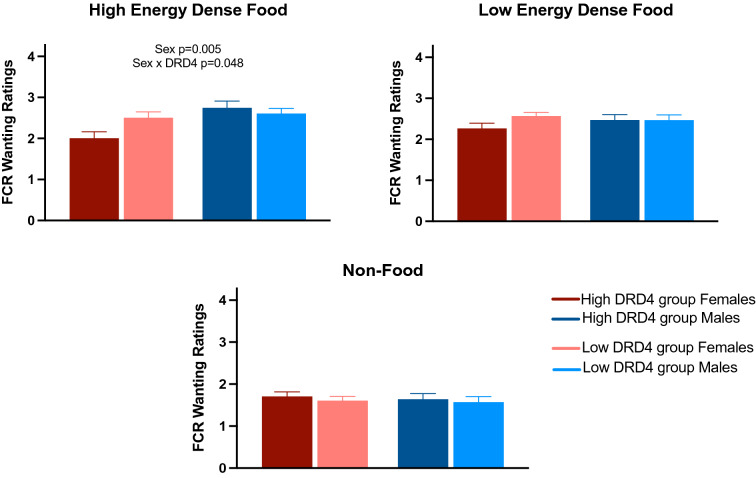
Food cue reactivity task wanting ratings (Mean ± SEM) in adolescents by predicted prefrontal DRD4 expression level (high vs. low) and sex (female vs. male).

Males reported significantly higher wanting ratings for the high-ED food cues (F(1,67) = 8.55, p = 0.005) relative to females. Wanting ratings for the high-ED food, low-ED food and non-food cues did not differ significantly by DRD4 group. There was a significant sex by DRD4 group interaction for the high-ED food cue wanting ratings, such that males in the high DRD4 group reported significantly higher ratings relative to females in the high DRD4 group, while males and females in the low DRD4 group had similar high-ED food cue wanting ratings (F(1,67) = 4.06, p = 0.048). FCR wanting ratings are depicted in Fig. 1.

### Imaging results

**Figure 2:**
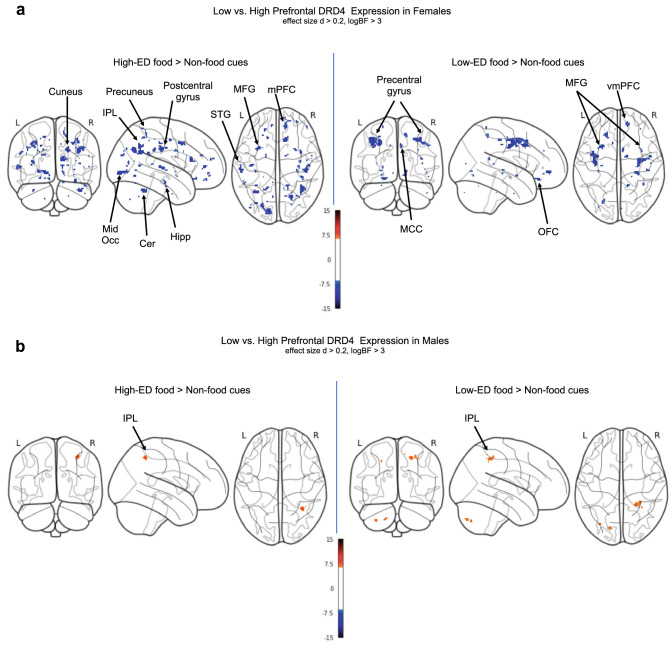
(**a**) Areas showing differential BOLD Response to High-ED Food vs. Non-Food and Low-ED Food vs. Non-Food Cues between Groups with Low vs. High Predicted Prefrontal DRD4 Expression among Adolescent Females. Figure depicts all clusters with effect size Cohen’s d > 0.2 and Bayes factor logBF > 3.0. Clusters surviving cluster extent threshold k > 10 are labelled. *IPL* inferior parietal lobule, *Mid Occ* middle occipital gyrus, *Cer* cerebellum, *MFG* middle frontal gyrus, *STG* superior temporal gyrus, *mPFC* medial prefrontal cortex, *Hipp* hippocampus, *vmPFC* ventromedial prefrontal cortex, *OFC* orbitofrontal cortex, *MCC* midcingulate cortex. (**b)** Areas showing differential BOLD Response to High-ED Food vs. Non-Food and Low-ED Food vs. Non-Food Cues between Groups with Low vs. High Predicted Prefrontal DRD4 Expression among Adolescent Males. Figure depicts all clusters with effect size Cohen’s d > 0.2 and Bayes factor logBF > 3.0. Clusters surviving cluster extent threshold k > 10 are labelled. *IPL* inferior parietal lobule.

In response to high-ED food compared with non-food cues, females in the low DRD4 group showed lower BOLD response in the cuneus, middle occipital gyrus, superior occipital gyrus, precuneus, inferior parietal lobule, superior temporal gyrus, postcentral gyrus, middle frontal gyrus, medial prefrontal cortex, hippocampus, and cerebellum. For the same contrast, males in the low DRD4 group showed greater BOLD response in the inferior parietal lobule relative to males in the high DRD4 group. For the contrast of low-ED food vs. non-food cues, females in the low DRD4 group showed lower BOLD response in the ventromedial prefrontal cortex (vmPFC), orbitofrontal cortex (OFC), precentral gyrus, middle frontal gyrus, midcingulate cortex, and superior parietal lobule relative to females in the high DRD4 group. For the same contrast, males in the low DRD4 group showed greater BOLD response in the inferior parietal lobule relative to males in the high DRD4 group. Results can be found in Table 2 and Figs. 2a,b.

**Table 2:** Areas showing differential BOLD Response to High-ED Food vs. Non-Food and Low-ED Food vs. Non-Food Cues in FCR task between groups with low vs. high predicted prefrontal DRD4 expression.

Contrast	Cluster size k	Peak Log odds (logBF)	X^a^	Y	Z
**Low > High DRD4 prefrontal expression**					
**FEMALE**					
**High-ED food > non-food cue**					
*n.s*					
**High-ED food < non-food cue**					
L Middle Occipital Gyrus	76	4.51	− 16	− 90	− 4
R Cuneus	39	4.95	20	− 66	30
R Cerebellum VI	37	4.35	38	− 50	− 32
L Middle Frontal Gyrus	28	4.01	− 30	12	42
L Inferior Parietal Lobule	28	4.08	− 34	− 58	40
R Postcentral Gyrus	28	4.47	44	− 22	34
L Superior Temporal Gyrus	25	3.82	− 60	− 20	2
R Medial Prefrontal Cortex	15	3.93	12	46	6
R Precuneus	14	3.32	14	− 48	60
R Hippocampus	13	3.90	32	− 16	− 20
R Middle Frontal Gyrus	11	3.39	38	6	44
L Superior Occipital Gyrus	11	4.03	− 16	− 86	24
**Low-ED food > non-food cue**					
*n.s*					
**Low-ED Food < Non-Food cue**					
L Precentral Gyrus	153	5.80	− 36	− 12	38
R Precentral Gyrus	91	5.13	36	− 8	46
L Middle Frontal Gyrus	56	4.57	− 26	14	42
R Middle Frontal Gyrus	23	3.46	36	8	46
R Midcingulate Cortex	19	4.82	6	12	38
R Ventromedial Prefrontal Cortex	19	5.31	14	50	− 4
L Orbitofrontal Cortex	19	3.74	− 18	34	− 16
R Superior Parietal Lobule	17	3.60	16	− 48	54
**MALE**					
**High-ED food > non-food cue**					
R Inferior Parietal Lobule	18	4.32	34	− 52	52
**High-ED food < non-food cue**					
*n.s*					
**Low-ED food > non-food cue**					
R Inferior Parietal Lobule	37	4.06	30	− 40	52
**Low-ED food < non-food cue**					
*n.s*					

Results adjusted for Population Stratification 1, Population Stratification 2 effect size = 0.2, logBF > 3, k >  = 10.

^a^Coordinates in MNI space.

## Discussion

Our results demonstrate that differential genetically-predicted DRD4 expression in the PFC is associated with differential patterns of brain activation in response to food cues of varying energy density (ED), when presented in the fed state. These differences appear to be sex dependent, such that adolescent girls with low predicted expression of DRD4 in PFC showed a pronounced pattern of lower activation to high-ED food cues across multiple brain regions implicated in diverse higher-order and lower-order cognitive processes including attention, memory, visual processing, taste processing and motor processing. Differences also vary somewhat by stimulus, such that adolescent girls with low predicted expression of DRD4 in the PFC showed lower activation of orbitofrontal cortex and ventromedial PFC, regions critical for valuation of stimuli, in response to low-ED cues. No sex by DRD4 interactions were apparent for ratings of internal state, or stimuli-related wanting during the FCR task. However, males reported more hunger and less fullness pre-scan compared to females, and adolescents with low DRD4 reported higher pre-scan boredom. Taken together, our results provide initial support for a model in which genetically-determined dopamine function in prefrontal cortex, and biological and social components associated with sex, act together to influence neural processing of food cues in the satiated state among adolescent girls and boys.

Our findings are broadly consistent with previous mechanistic and clinical studies on function of the prefrontal cortex and its modulation by dopamine through development. Specifically, the prefrontal cortex is known to modulate behaviors relevant to eating behavior and obesity via anatomical and functional connections with other cortical and sub-cortical brain areas^8,9^. Further, during adolescence the process of cortical neuronal maturation and synaptic neurotransmission has been found to be dopamine dependent^10,48,49^. Altered expression of dopamine receptor genes could therefore be associated with dysregulated activity in cortical ensembles, affecting the capacity for cortical inhibition and thereby influencing the functioning of neural circuits underlying food preference and selection related behaviour^50–52^. The differentiation of our neuroimaging association results by sex is also broadly consistent with a substantial literature demonstrating sex differences in the rate of structural and functional maturation of prefrontal cortex during adolescence^30^ as well as in neural and behavioral responses to food cues^31,32^.

Against this general background, certain features of our results merit further discussion. First, we found much more evidence for differentiation in neural activation patterns among girls, as opposed to boys, with relatively low predicted DRD4 expression. Specifically, girls with low predicted expression of DRD4 in the PFC showed a pronounced pattern of reduced activation to high-ED food cues across a widespread appetitive circuit of brain regions implicated in diverse processes including attention, memory, visual processing, taste processing and motor processing. This suggests that girls with relatively reduced dopamine receptor 4 (DRD4) expression in the PFC may exhibit a relative blunting, or suppression, of appetitive processing in the brain in the context of a task requiring evaluation of subjective appetite in response to high-calorie stimuli. In contrast, girls with relatively high DRD4 expression may be most likely to mount a complex response to a highly palatable food stimulus in the environment, engaging multiple regions associated with stimulus salience, motivation, and attentional control. The emergence of these differential patterns of activation among girls, but not boys, is consistent with greater likelihood of a complex appetitive response among girls. The behavioral consequences of differentiation according to prefrontal DRD4 expression cannot be inferred within this cross-sectional study. However it was notable that mean wanting ratings for high-ED foods for girls with low expression (2.5) were more similar to the higher ratings among boys with either low (2.7) or high (2.8) expression, than for girls with high expression (2.0). Larger longitudinal studies may be able to determine whether low PFC DRD4 expression in girls could in some individuals or settings act to increase obesity risk by decreasing complex processing of high-ED foods, or, alternatively, to increase obesity resilience by decreasing the salience of high-ED foods during evaluation of appetite.

Also striking was the differentiation we observed between patterns of activation in response to high-ED and low-ED foods compared to non-food in the case of girls. For girls, group differences in activation were largely distinct for each stimulus category, with the exception of the middle frontal gyrus, which showed relatively reduced activation bilaterally in response to both high-ED and low-ED foods in comparison with non-foods. Since the middle frontal gyrus plays a role in reorienting attention from externally-driven to endogenous attentional control^53^, diminished activation here may reflect a relatively reduced inclination to attend to internal satiety signals when evaluating the desire to eat both higher and lower energy foods. However, the diminished response to high-ED foods across a much broader array of regions implicated in motivation and attention suggests that evaluating appetite for high-energy foods engages a much more complex neural response than the same process for low-ED foods, among girls with lower DRD4 expression in PFC. In contrast, boys with lower predicted DRD4 expression showed relatively increased activation of right inferior parietal lobe for both high-ED and low-ED foods compared with non-foods. This result was in contrast to results in females, who showed relatively decreased activation of left inferior parietal lobule in response to high-ED foods vs. non-foods together with decreased activation across a large number of attentional processing regions. Given the IPL’s role in maintaining attentional control on current task goals while simultaneously preparing a response to salient stimuli in the environment^54^, this phenomenon suggests that decreased PFC dopamine function may act to increase simple attention toward foods compared with non-foods in boys, and to decrease more complex attentional processing specifically toward high-ED foods in girls.

Also of note was that adolescent girls with lower DRD4 expression showed lower activation of vmPFC and OFC in response to low-ED compared with non-foods. Both vmPFC and OFC have been implicated in goal directed behaviours especially in determining the value of a goal such as food reward during the process of decision making^55,56^. Interestingly, neuronal activity in the vmPFC during evaluation of value has been found to be irrelevant to the degree of self-control executed by participants^57^. Studies of neural responses to food cues have also shown activation of the OFC among healthy-weight adults, consistent with involvement in neural representation of value^58–61^. The DRD4 group difference we saw here may therefore reflect a relatively decreased valuation of healthy low-ED foods among girls with lower dopamine function in PFC.

While our imaging analyses revealed diverging effects of DRD4 expression in females and males, our behavioral analyses showed main effects of sex on appetite ratings, and of DRD4 on ratings of emotion, namely boredom. Regardless of predicted PFC DRD4 group, adolescent males reported greater pre-scan hunger, lower pre-scan fullness, and higher wanting ratings for the high-ED food cues, compared with adolescent girls. This is consistent with other evidence for enhanced food motivation in men compared with women throughout the lifespan, and especially during puberty and adolescence when sex differences in energy demands become pronounced^62,63^. In our data it therefore appears that the effect of sex generally trumps that of DRD4 expression to influence subjective appetite. Since our goal for the current work was to explore effects of DRD4 expression within each sex rather than to test main effects of sex, and DRD4 status did not significantly influence appetite reports in either sex, we here present our results unadjusted for internal state ratings. It was notable, however, that adolescent girls with low predicted DRD4 showed wanting ratings for high-ED foods (and to some degree wanting ratings for low-ED foods and pre-scan hunger and desire to eat) that were more similar to those of adolescent boys than to their peers with high predicted DRD4, suggesting that behavioral indices of appetite in this group may also show some evidence for heightening. Since the current investigation was a secondary analysis, our cell sizes for these comparisons were small and no a priori power calculation was conducted to generate the target sample size. It is therefore possible that a larger study would be more sensitive to such effects. Future research could also consider repeating analyses with control for baseline appetite to distinguish whether observed results are substantially attributable to effects of predicted DRD4 on global appetite, or to effects of predicted DRD4 on neural food cue responses independent of general subjective appetite. Potential influence of PFC DRD4 expression on emotion-driven eating should also be investigated further. Our fMRI task paradigm for this study was not designed to explicitly examine the effect of emotion on neural food cue response. However, consistent with the role of dopamine in motivation, we found that adolescents with low predicted PFC DRD4 expression expressed higher levels of boredom before beginning the task, and boredom has been shown in some individuals to trigger consumption of highly palatable snack foods^64^.

Our results suggest that prefrontal DRD4 expression and sex may influence neural and behavioral food cue responses, and that prefrontal DRD4 expression may show unique impacts on these outcomes among adolescent girls. One potential explanation for the divergence of our results by sex may be sex differences in relative stages of brain development rather than enduring sex differences persisting through development^65^. For example, brain circuits subserving executive function, including the PFC, are relatively late-maturing in comparison with other brain regions, and mature more rapidly in females^66^. Variability in the functioning of brain regions engaged during decision making may therefore be more pronounced in girls, both mediated by dopamine function, and allowing differences driven by dopamine function to emerge. It would therefore be of interest to test whether these sex differences are also apparent in adulthood, when both sexes have reached the same level of maturation. A related potential explanation is hormonal. Hormonal transition periods such as adolescence are marked by structural and functional maturation of cortical networks and increased vulnerability to behavioral disorders involving dopamine function^8^, and girls in our sample were more advanced in pubertal stage. Further, dopamine function is influenced by sex and previous evidence shows a differential role of sex hormones such as insulin, leptin, progesterone and estrogen on dopamine-regulated behaviours including motivation, reward and emotion processing^63,67,68^. Estrogen levels have been proposed to modulate brain regions subserving emotional regulation^69,70^. Emotional eating behavior may also be regulated by estrogen through estrogen receptor-alpha (ERalpha)^71^. Other mechanisms by which sex hormones could be regulating emotional eating behaviours include gastric mechanoreceptors, glucagon-like peptide-1 (GLP-1), gustatory sensations and orosensensory hedonics^72^. Estrogen has been proposed to facilitate dopaminergic neurotransmission in cortical and sub-cortical brain regions^73,74^. Estrogen may also facilitate glutamatergic and suppress GABAergic inhibitory transmission through its action at GABAA receptors^75^. A PET study conducted during the postpartum period (a period with increased hormonal and emotional dysregulation) found reduced levels of estrogen to be associated with increased levels of an enzyme involved in metabolising dopamine in the female brain^76^. Future studies examining the impacts of puberty are warranted to further investigate hormonal effects on cortical dopaminergic transmission and its effect on food cue response. Given evidence that neural food cue responses vary with menstrual cycle in adult women^77–79^, studies comparing young girls in specific phases of the menstrual cycle may also help to clarify the current findings. It should also be noted that the sex differences by DRD4 expression that we observed here could in part be attributable to social as well as hormonal forces such as increased perceived pressure for girls to display prosocial behaviors^80^, or to restrict dietary intake and attain a low body weight^69,72^. Such environmental factors could result in a more complex, conflicting cognitive processing of food cues in girls which is selectively minimized among girls who show a blunted response to environmental stimuli due to decreased genetically-driven DRD4 function in PFC.

The present results should be considered in the context of an emerging literature demonstrating that genetically-influenced dopamine function can modulate the effects of sex, and environmental factors, on variables associated with obesity risk^25,81,82^. For example, a sex by DRD2 rs6722 genotype interaction was previously observed such that females carrying the TT allele performed significantly better on an attention task than males carrying the CC allele^82^, while another study found that the VNTR DRD4-7R-hypofunctional allele was more strongly associated with high caloric food intake in young girls than boys^83^. Despite evidence in the same cohort that DRD4 expression interacted with SES and to influence food intake^20^, exploratory analyses did not support interactive effects of SES and DRD4 group on neural responses to food cues. However, as previously discussed, the sex-specific effects we observed are consistent with environmental, as well as biological, correlates of sex modulating effects of DRD4 expression, supporting the overall model that DRD4 expression modulates environmental effects on obesity risk.

Limitations of our study include the sample size. For example, studies with considerably larger sample sizes would be required to investigate potential three-way interactions between DRD4 expression, sex, and socioeconomic variation. Also, our comparisons by sex and DRD4 group were not able to show that sex and predicted DRD4 expression interacted to influence food cue wanting ratings, or BMI z score. This suggests that the brain activation effects we observed may be more proximally and therefore strongly related to the effects of DRD4 expression than downstream behavioral or anthropometric outcomes. Since perceptual uncertainty influences decision making and is dependent on dopamine^84^, and reactivity to cues is predicted by the value of the reward and its availability in the given environment^85^, neural responses within our food cue task, which centered on unavailable rewards, were likely most optimal for capturing effects of DRD4 expression. Another limitation was that we did not investigate potential social and environmental variables that could explain the sex differences we observed. Nevertheless, taken in the context of complementary research, our results support sex-specific effects of prefrontal DRD4 on brain food responsiveness in adolescence, with modulation of appetitive circuit responses to food cues most apparent in female adolescents.

## Data Availability

The datasets used for the current study are available from the corresponding author on reasonable request.
